# Optical DNA Biosensor Based on Square-Planar Ethyl Piperidine Substituted Nickel(II) Salphen Complex for Dengue Virus Detection

**DOI:** 10.3390/s18041173

**Published:** 2018-04-12

**Authors:** Eda Yuhana Ariffin, Ling Ling Tan, Nurul Huda Abd. Karim, Lee Yook Heng

**Affiliations:** 1 School of Chemical Sciences and Food Technology, Faculty of Science and Technology, University Kebangsaan Malaysia, Bangi 43600, Selangor D.E., Malaysia; edayu_hanna@yahoo.com (E.Y.A.); nurulhuda@ukm.edu.my (N.H.A.K.); leeyookheng@yahoo.co.uk (L.Y.H.); 2 Southeast Asia Disaster Prevention Research Initiative (SEADPRI-UKM), Institute for Environment and Development (LESTARI), Universiti Kebangsaan Malaysia, Bangi 43600, Selangor D.E., Malaysia

**Keywords:** dengue virus detection, nickel(II) salphen complex, optical DNA biosensor, porous silica nanospheres, reflectance measurement, synthetic DNA binder

## Abstract

A sensitive and selective optical DNA biosensor was developed for dengue virus detection based on novel square-planar piperidine side chain-functionalized *N*,*N*′-bis-4-(hydroxysalicylidene)-phenylenediamine-nickel(II), which was able to intercalate via nucleobase stacking within DNA and be functionalized as an optical DNA hybridization marker. 3-Aminopropyltriethoxysilane (APTS)-modified porous silica nanospheres (PSiNs), was synthesized with a facile mini-emulsion method to act as a high capacity DNA carrier matrix. The Schiff base salphen complexes-labelled probe to target nucleic acid on the PSiNs renders a colour change of the DNA biosensor to a yellow background colour, which could be quantified via a reflectance transduction method. The reflectometric DNA biosensor demonstrated a wide linear response range to target DNA over the concentration range of 1.0 × 10^−16^–1.0 × 10^−10^ M (R^2^ = 0.9879) with an ultralow limit of detection (LOD) at 0.2 aM. The optical DNA biosensor response was stable and maintainable at 92.8% of its initial response for up to seven days of storage duration with a response time of 90 min. The reflectance DNA biosensor obtained promising recovery values of close to 100% for the detection of spiked synthetic dengue virus serotypes 2 (DENV-2) DNA concentration in non-invasive human samples, indicating the high accuracy of the proposed DNA analytical method for early diagnosis of all potential infectious diseases or pathological genotypes.

## 1. Introduction

Dengue fever has become a major public health concern in Malaysia and many other tropical countries [1]. The dengue virus exists as four serotypes, namely DENV-1, DENV-2, DENV-3 and DENV-4. These four subtypes of dengue viruses have been identified in more than one hundred countries, and the global incidence of dengue cases was estimated to be between 50 and 100 million infections a year. DENV-2 is mainly responsible for the severe dengue illnesses in Southeast Asia [2]. In Malaysia, this arthropod-borne virus is endemic. Dengue viruses are transmitted to humans through the bites of the infected female Aedes mosquitoes, especially Aedes aegypti and Aedes albopictus [3]. The symptoms of dengue fever include high fever, body rashes, headache, sore throat, vomiting and body pain, which includes joint and muscle pain. Dengue hemorrhagic fever or dengue shock syndrome is the main arboviral disease caused by the dengue virus in humans and it is taken seriously as it is fatal in the worst-case scenario, where a sharp drop in the patient’s blood platelets is followed by internal bleeding.

Recognition of shock in its early stages with prompt fluid replacement therapy can result in a good clinical outcome and prevent mortality. However, laboratory confirmation of dengue infection relies on serological testing, which is time-consuming as the patient is required to produce a detectable level of anti-dengue antibodies, which normally takes some four to five days to yield a positive reactive result [4]. The late diagnosis often results in delayed treatment, which can be less effective in preventing or decreasing further complications. Furthermore, serology tests often result in cross-reactivity with other flaviviruses e.g., West Nile virus, Zika virus, Japanese encephalitis and yellow fever. Therefore, a simpler, faster and more specific detection method is needed for the diagnosis of this pathogenic arbovirus.

The development of a rapid and high-throughput reverse transcription-polymerase chain reaction (RT-PCR) method allows sensitive detection of virus-specific nucleic acid including RNA in human serum. However, it is difficult to get truly quantitative results using this method, and interpretation difficulties ascribed to false positive amplification often occur, where the product has the approximate expected length. Real-time PCR (TaqMan) and PCR-ELISA, on the other hand, are technically highly sensitivity and specific and could minimize the risk of cross contamination [5,6]. Nevertheless, the number of amplicons detected is restricted by the number of fluorophores, variation increases with cycle number and wide deployment is expensive [7,8]. 

To date, dengue viral diagnostic technologies are too complex, time-consuming and expensive to implement. Cheng et al. [9] for instance, developed a nanoporous alumina membrane-based biosensor for the detection of dengue virus particles. The coating of the nanoporous alumina membrane on the platinum electrode surface required physical sputtering followed by electrochemical anodization procedure. However, the selectivity of this biosensor towards other non-specific viruses’ particles remains questionable. In order to improve the selectivity of the biosensor towards the dengue virus, DNA is an ideal target for specific detection of viral pathogens. Zhang and co-workers [4] have constructed an innovative field-effect transistor (FET)-based DNA biosensor for highly sensitive and rapid detection of the dengue virus. However, the fabrication of the silicon nanowire transducer required etching and oxidation of a silicon-on-insulator wafer at high temperature, and the tedious need for further lithographic methods. Another electrochemical DNA-based biosensor for the detection of dengue serotypes at picomolar concentration level was fabricated using gold nanoparticles-polyaniline hybrid composites. The DNA hybridization was characterized by electrochemical impedance spectroscopy (EIS) and cyclic voltammetry (CV) [1]. However, one of the most challenging disadvantages of electrochemical biosensor detection methods is signal reduction from fouling agents and interference from chemicals present in the sample matrix.

In order to eliminate electrical interference and the need for professional handling skills, an optical DNA biosensor is an alternative to the conventional molecular biology techniques and electrochemical methods [10]. Optical biosensors have attracted the attention of many researchers because of their small size, ease of operation and freedom from electrical induced noise [11,12,13]. The principle of optical sensing is typically based on the interaction between a sample and light. The type of optical transduction element (i.e., absorption spectroscopy, fluorescence spectroscopy or reflectance spectroscopy) used depends on the optical characteristics of the chemical reagent and physical properties of the immobilization matrix used. Reflectance spectrometry has been shown to be useful for quantitative determination of small amounts of pollutants. Nevertheless, the precision of reflectance spectrometry measurements cannot be better than 10%, thus, reflectance spectroscopy is considered qualitative and non-reproducible due to the effect of the inhomogeneous media. However, with the development of new techniques employing optical fiber or reflectance spheres has the situation has improved significantly [14].

In this work, ethyl piperidine substituted *N*,*N*′-bis-4-(hydroxysalicylidene)-phenylenediamine-nickel(II) or nickel(II) salphen complex with piperidine side chain, a synthetic optical oligonucleotide label was synthesized via a facile one-pot reaction. Salphen complex, also known as *N*,*N*′-bisphenylene(salisilidine imine) is a Schiff base ligand that possesses N_2_O_2_-donor atoms, which can form coordinate (dative covalent) bonds with metal ion at its centre through a ligand-to-metal charge transfer (LMCT) reaction by donating a lone pair of electrons from the respective donor groups to the orbital *d* of the metal ion. The metal ion is in the plane formed by the four metal-ligating N_2_O_2_-donor atoms, and the metal complex has square-planar geometry that permits it to intercalate within a DNA duplex by aromatic stacking interaction [15]. As the proposed yellow coloured Schiff base metal complex gave a colour change during the probe to target DNA hybridization reaction, an optical dengue virus DNA biosensor was developed based on light reflection transduction to quantify the corresponding targeted DNA concentration. Glutaric acid, the organic dicarboxylic acid linker was employed to attach the aminated DNA probe onto the porous silica particles (PSiNs) surface by amide covalent bonding. The resulting solid-state DNA biosensor allowed visual detection of dengue viruses by the human naked eye simply by visual observing the colour change at the surface of the PSiNs-based DNA biosensing platform.

## 2. Materials and Methods

### 2.1. Chemicals

3-Aminopropyltriethoxysilane (APTS, 98%), glutaric acid (GA), 1-(2-chloroethyl)piperidine hydrochloride, 2,4-dihydroxybenzaldehyde and synthetic oligonucleotides 100 μM (Table 1) were supplied by Sigma-Aldrich. Dimethyl sulfoxide, nickel(II) acetate tetrahydrate [Ni(OAc)_2_•4H_2_O], *N*,*N*′-dimethylformamide (DMF), ethyl acetate, chloroform and n-hexane were obtained from ChemAR. 1,2-diamino benzene, diethyl ether and potassium carbonate were purchased from Merck. Tetraethyl orthosilicate (TEOS, 98%) and Tween 20 surfactant were procured from Fluka. Sodium fluoride (NaF, 98.5%) and methanol (99.8%) were obtained from R & M Chemicals and JT Baker, respectively. Stock solution of DNA probe was prepared in 0.05 M K-phosphate buffer (pH 7.0), whilst complementary DNA (cDNA) solution was prepared in 0.05 M K-phosphate buffer containing 0.1 M KCl at pH 7.0. Milli-Q water was used for all aqueous solutions preparation.

### 2.2. Instrumentation

^1^H and ^13^C Nuclear Magnetic Resonance (NMR) [1-dimensional (1D) and 2-dimensional (2D)] spectra were recorded on a Bruker/AVANCE III 600 MHz Fourier Transform Nuclear Magnetic Resonance 400 MHz cryoprobe spectrometer using tetramethyl silane (TMS) as an internal standard and deuterated dimethyl sulfoxide (DMSO-*d*_6_) solvent. Fourier transform-infrared spectroscopy (FTIR) spectra were captured on the Perkin Elmer Spectrum 400 FT-IR in the wavenumber range of 4000–400 cm^−1^. Mass spectra were acquired from BRUKER MicroTof-Q with DIONEX Ultimate 3000 LC. Differential Scanning Calorimeter (DSC, DSC882e Mettler Teledo) was used to measure the metal salphen complex melting temperature. The binding interaction between metal complex and double-stranded DNA (dsDNA) was carried out by spectrophotometric titration method using Varian Cary^®^ 50 UV-Vis Spectrophotometer. The size distribution and particle morphology of the as-synthesized porous silica nanospheres (PSiNs) were examined by field emission scanning electron microscopy (FESEM, JEOL Co. Model JSM-6700F, Japan). A fiber optic reflectance spectrometer (SD 2000 Ocean Optics) was employed to record the reflectance intensity of the DNA biosensor in the wavelength range of 200–1099 nm.

### 2.3. Synthesis of Nickel(II) Salphen Complex with Piperidine Side Chain

The nickel(II) salphen complex functionalized with piperidine side chain was synthesized according to a method reported by Vilar et al. [16]. In brief, 1,2-diamino-benzene (0.360 g, 2.5 mmol) was reacted with 2,4-dihydroxybenzaldehyde (0.699 g, 5.0 mmol) in ethanol (50 mL) under reflux at 80 °C for 30 min. Ni(OAc)_2_•4H_2_O (1.24 g, 5.0 mmol) was then added, and the chemical reaction was heated again under reflux for another 24 h. The reaction mixture was then cooled to room temperature, and the dark brown precipitate was filtered and washed sequentially with ethanol (100 mL), diethyl ether (50 mL) and Milli-Q water (50 mL) to obtain the nickel(II) salphen complex as dark brown solid. The as-synthesized nickel(II) salphen complex (0.409 g, 1.0 mmol) and 1-(2-chloroethyl) piperidine hydrochloride (0.921 g, 5 mmol) were then dissolved in 60 mL of DMF in the presence of potassium carbonate (0.704 g, 8 mmol) and heated under reflux at 80 °C for 72 h. After that, the solvent was evaporated under reduced pressure by using a rotary evaporator. The resulting dark red solid was later recrystallized by means of DMF and diethyl ether solvent-pair recrystallization at the volume ratio of 1:3 (DMF:diethyl ether) to yield the desired piperidine side chain functionalized nickel(II) salphen complex.

### 2.4. Synthesis of Aminated Porous Silica Particles

A homogeneous mixture of TEOS and 0.02 M Tween 20 at the molar ratio of 8:1 and NaF salt was obtained by continuous stirring on a magnetic stirrer (IKA 9500100 C-Mag HS7) for three and half hours. After that, the solution was incubated for a day in an incubating shaker at room temperature (27 °C). The resulting white precipitate was then centrifuged at 4000 rpm for 15 min and sequentially washed with abundant ethanol and Milli-Q water. The white slurry obtained was later dried and calcined in air at 200 °C for 6 h followed by 620 °C for another 6 h to produce the PSiNs. About 2 mL of APTS was added to 100 mg of PSiNs and allowed to react overnight via continuous stirring on the magnetic stirrer at ambient conditions. Subsequently, the resulting white slurry of APTS-modified PSiNs was air dried for 24 h.

### 2.5. Fabrication of PSiNs-Based Optical DNA Biosensor

**Figure 1:**
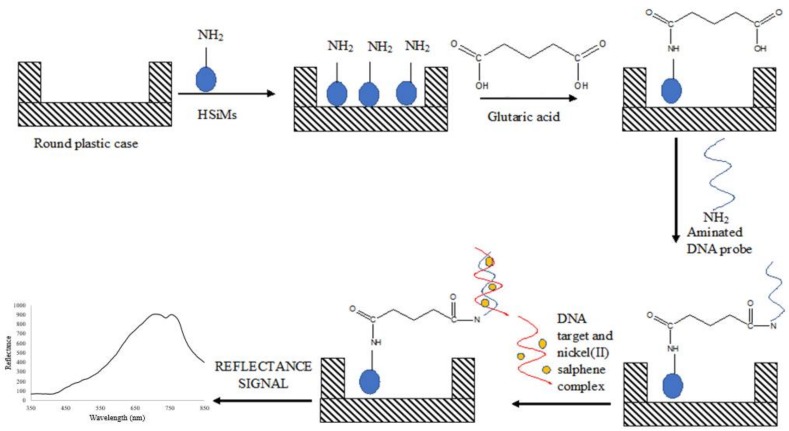
The schematic diagram representing the development of reflectance dengue virus DNA biosensor based on the use of optical Schiff base metal salphen complex synthetic DNA label.

Some 50 mg of dried APTS-modified PSiNs particles prepared from Section 2.4 were dispersed in 500 µL of ethanol and sonicated for about 5 min to produce a PSiNs suspension at 0.1 mg/µL (i.e., 50 mg/500 µL ethanol). About 50 µL of PSiNs suspension was then deposited in a round plastic supporting case by using a micropipette. As 500 µL of suspension consists of 50 mg of PSiNs particles, then 50 µL of PSiNs suspension consists of about 5 mg of PSiNs particles. The diameter of the round cap of 500 μL Eppendorf microcentrifuge tube is 5 mm and its height is 3 mm. Glutaric acid (GA, 1 g/16 mL Milli-Q water) at 80 µL was then added and left for reaction for an hour. The excess GA was then washed away by using 0.05 M K-phosphate buffer (pH 7.0). Next, about 80 µL of 4 µM dengue DNA probe was dispensed onto the GA-modified PSiNs and left overnight at 4 °C to allow covalent immobilization of the DNA probe to take place. The unbound DNA probe was later eliminated by rinsing with copious amounts of the 0.05 M K-phosphate buffer at pH 7.0. DNA hybridization was carried out by dropping 80 µL of complementary DNA (cDNA) solution at 1 × 10^−7^ µM containing 1 mM nickel(II) salphen complex onto the DNA probe-immobilized PSiNs surface and allowed to react for 2 h. The loosely bound target DNA and free metal complex intercalator were removed by washing the PSiNs-based DNA biosensor with abundant 0.05 M Na-phosphate buffer and left to dry for 30 min. The reflectance intensity of the DNA biosensor before and after reaction with the target DNA was measured with a fiber optic reflectance spectrophotometer. The stepwise process for the fabrication of optical dengue virus DNA biosensor using nickel(II) salphen complex as DNA hybridization marker is shown in Figure 1.

### 2.6. Characterization and Performance Evaluation of Reflectance Dengue Virus DNA Biosensor

Effect of PSiNs loading was carried out by varying the volume of PSiNs suspension loading between 80 µL and 140 µL in the plastic case prior to GA modification and DNA probe immobilization. The effect of DNA probe concentration was investigated by immobilizing various DNA probe concentrations from 0.5 to 4.0 µM on 8 mg PSiNs and 1 mM metal complex marker. The DNA marker concentration effect was conducted by changing the transition metal complex concentration from 0.5 mM to 5 mM, whilst DNA probe loading was fixed at 2.5 µM. For buffer capacity effect, Na-phosphate buffer in the concentration range of 0.04–0.10 M at pH 7.0 was used as the DNA hybridization medium towards cDNA detection at 1 × 10^−7^ µM in the presence of 2 mM nickel(II) salphene complex with piperidine side chain marker. The effect of pH on the reflectance DNA biosensor response was studied by altering the 0.07 M Na-phosphate buffer pH between pH 5.0 and pH 8.0 in the determination of 1 × 10^−7^ µM target DNA containing 2 mM metal complex label. In order to examine the DNA hybridization time, 1 × 10^−7^ µM cDNA containing 2 mM optical metal complex DNA label was allowed to react with the immobilized DNA probe from 30 min to 210 min in 0.07 M Na-phosphate buffer at pH 7.0. The DNA biosensor shelf life study was performed by preparing 50 units of PSiNs-based DNA biosensors and storing at 4 °C, and three units of DNA biosensors were tested intermittently with 1 × 10^−7^ µM cDNA and 2 mM metal complex marker at pH 7.0 throughout the three weeks of the experimental period. The dynamic linear response range of the DNA biosensor was determined by using various cDNA concentration between 1 × 10^−12^ µM and 1 × 10^−2^ µM with constant nickel(II) salphen complex concentration at 2 mM. DNA biosensor selectivity was studied with target DNA hybridization, non-target DNA and 1-base unpaired DNA at 1 × 10^−8^ µM under optimum DNA hybridization conditions. Finally, the optimized DNA biosensor was used for recovery of spiked synthetic DENV-2 DNA (at 1 × 10^−7^ µM, 1 × 10^−5^ µM and 1 × 10^−4^ µM) in human saliva and urine samples collected from three healthy volunteers in the sterilized plastic bottles without any pre-treatments.

## 3. Results and Discussion

### 3.1. Characterization of Piperidine Side Chain-Modified Nickel(II) Salphen Complex

**Figure 2:**
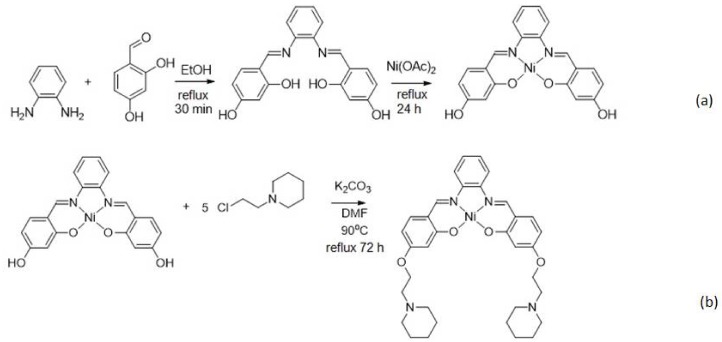
The chemical reactions involved in the synthesis of ethyl piperidine substituted nickel(II) salphen complex: (**a**) Chemical reaction between 1,2-diamino-benzene and 2,4-dihydroxybenzaldehyde to form the salphen ligand followed by reaction with Ni(OAc)_2_•4H_2_O to form the nickel(II) salphen complex; (**b**) The nickel(II) salphen complex further reacted with 1-(2-chloroethyl) piperidine hydrochloride in the presence of potassium carbonate to obtained the desired piperidine side chain functionalized nickel(II) salphen complex.

We have previously synthesized an unsubstituted Schiff base complex based on Zn^2+^ ion, and the low solubility characteristic of the metal complex in the water environment hindered the resulting DNA biosensor from being directly applied in the human bodily fluid samples [17]. Figure 2 represents the chemical reactions involved in the formation of ethyl piperidine grafted nickel(II) salphen complex. The brownish red nickel(II) salphen complex’s yield was obtained at 77% (794.2 mg). As the unsubstituted nickel(II) salphen complex has very poor water solubility, alkylamine substituent such as ethyl piperidine was introduced to enhance the metal complex’s solubility in an aqueous environment. The alkylamine substituent is known to be protonated at physiological pH (i.e., at pH 7.4) and this literally enhances the solubility of compounds and DNA affinity [18]. The resulting nickel(II) salphen complex was functionalized with ethyl piperidine moieties in a SN_2_ reaction in the presence of K_2_CO_3_. Phenolic -OH of the nickel(II) salphen complex was reacted with the chloroalkyl chain to form the attachment of cationic substituent to the aromatic rings of the complex to obtain the desired piperidine side chain-modified nickel(II) salphen complex with a relatively good yield (626.0 mg, 68%). It should be noted that the synthesis of the complex has been reported with different applications [16].

^1^H NMR spectroscopic analysis of the ethyl piperidine substituted nickel(II) salphen complex displayed all the expected signals with the right integration and multiplicities (Figure S1a). The DMSO-*d*_6_ solvent peak appeared at 3.45 ppm. About 11 proton peaks were observed because of the symmetrical structure of the complex molecule. The formation of the Schiff base ligand is exhibited by the presence of a singlet at 8.60 ppm, which is the characteristic of imine proton. The disappearance of the aldehyde proton peak at 9.93 ppm corresponds to the full conversion of 2,4-dihydroxybenzaldehyde starting material to form the N=C-H imine protons [19]. Grafting of the amine side chain to the metal salphen complex was evidenced by the proton chemical shifts corresponding to the ethyl piperidine functional group at around 1.36–4.10 ppm with the correct integration versus aromatic signals. Figure S1b in the Supplementary Material shows the ^13^C NMR spectrum of ethyl piperidine functionalized nickel(II) salphen complex. DMSO-*d*_6_ solvent was detected at the chemical shift of 39 ppm. It was expected that the symmetrical metal salphen complex containing 17 carbons would give chemical shift signals in the carbon NMR, however, only 15 peaks were observed due to the overlapping signal at chemical shifts of 54.82 ppm and 25.99 ppm. The presence of five peaks at the aliphatic region of the ^13^C NMR spectrum showed the expected signals correspond to the ethyl piperidine side chain. Two-dimensional proton NMR (COSY and HMQC) spectrum (not shown for brevity) reveals that there was no interaction between H_3_ and the adjacent hydrogen atoms (i.e., H_1_, H_2_, H_4_, H_5_ and H_6_) that were bonded to the benzene group.

The FTIR spectrum for nickel(II) salphen complex with piperidine side chain is shown in Figure S2. The absorption bands at 1122 cm^−1^, 2859 cm^−1^ and 2933 cm^−1^ are associated with the C-O, C-HO-, and CH_2_- functional groups, respectively. The absorption band for C=N stretch is observed at 1606 cm^−1^ and C=C aromatic stretching bands are determined at 1454 cm^−1^ and 1570 cm^−1^. Mass spectrometry (ESI-MS) analysis (Figure S3) confirmed that the ethyl piperidine grafted nickel(II) Schiff base complex was successfully synthesized with the molecular peak obtained at 627.2 a.m.u, which is consistent with the theoretical molecular weight for C_20_H_14_N_2_NiO_4_ at 627.4 g mol^−1^. The molecular-ion peaks appeared at *m*/*z* 314.12, 516.13 and 725.21 correspond to [M+Na]^+^. Na^+^ ions were present in the mass spectrum because they were produced from the mass spectrometer instrument using electron spraying ionization mode. The purity of Schiff base metal salphen complex was determined by DSC with a melting temperature of 249 °C.

### 3.2. UV-Vis Titration Studies to Evaluate the Interaction between Nickel(II) Salphen Complex and DNA

UV−vis spectroscopic titration was carried out in order to obtain the binding constant of the complex toward double stranded DNA (dsDNA) and to gain insight into the binding mode of this complex. Information on the binding mode is useful in determining the suitability of the complex as a DNA hybridization indicator. In general, a coordination complex with metal at its centre would render square-planar geometry shape of the metal complex that binds strongly to the dsDNA through an intercalation mechanism [20]. Figure S4 in Supplementary Material shows the UV-Vis absorption spectra for titration of the ethyl piperidine substituted nickel(II) salphen complex with dsDNA. The UV-V spectra showed strong absorption at 367 nm associated with intra-ligand π−π* transitions and 425 nm, which involved both the ligand and the metal centre associated with d-d transition. Upon addition of the dsDNA to the Schiff base metal salphen complex, it resulted in hypochromism, whereby some 17% decrement in maximum absorption peak of the metal complex between 360 and 380 nm was observed. In addition, a noticeable red-shift, about 7 nm was also perceived. The hypochromicity and bathochromicity occurred due to the reduction in the energy gap between HOMO and LUMO molecular orbitals [21].

The intrinsic binding affinities toward dsDNA and CT-DNA were determined by monitoring changes in complex absorption intensities upon increasing the DNA concentration. The Scatchard plot in Figure S5 in the Supplementary Material shows a linear function that implies that there is one type of binding site for the complex under study since linear Scatchard plots are suggestive of ligand binding to equivalent and independent binding sites. By using the Scatchard equation (the plot of D/Δεap versus D), the binding constant (K) value of the metal salphen complex towards dsDNA was estimated at K = 3.87 × 107 M^−1^, which indicates a moderate interaction of the nickel(II) complex with dsDNA. Similar binding affinity values were also reported for other dsDNA intercalators [22].

According to Topala et al. [23], as the metal complex binds to the DNA via intercalation reaction, its shows hypochromism and bathochromism or hyperchromism, which is attributed to electrostatic interaction, hydrogen bonding and groove binding (minor or major) along the outside of the DNA double-helical structure. Therefore, it is presumed that the as-synthesized piperidine side chain functionalized nickel(II) salphen complex was bound to the dsDNA by intercalation between base pairs ascribed to the square-planar coordination geometry structured of the Schiff base ligand bonded to the metal centre that enhances its ability to intercalate between dsDNA [16].

### 3.3. DNA Biosensor Based on APTS-Modified Porous Silica Nanospheres

**Figure 3:**
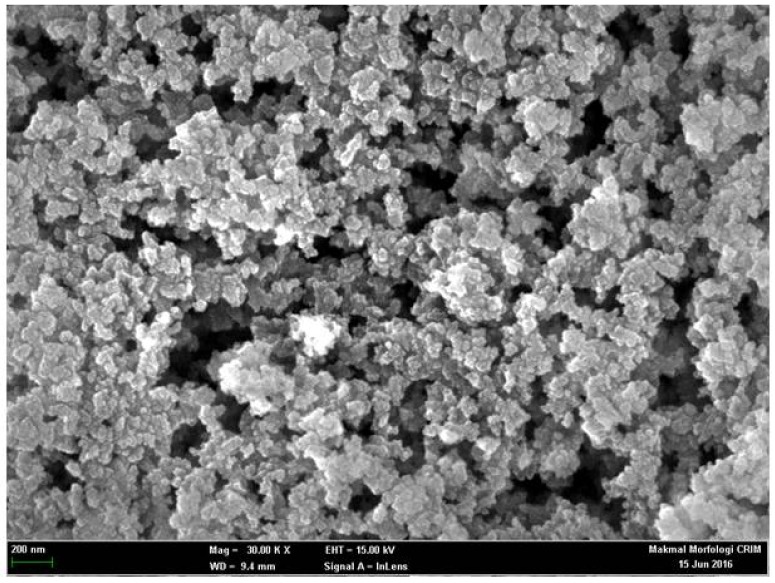
FESEM micrograph of the as-synthesized APTS-functionalized silica particles captured by JEOL FESEM at 15 kV acceleration voltage and 30 kX magnification.

The FESEM micrograph shows the silica particles are spherical in shape with rough surfaces and are densely arranged (Figure 3). The APTS-functionalized PSiNs are particles between 50 nm and 80 nm in size. The synthesis of PSiNs based on a sol-gel process is based upon the hydrolysis and condensation or cross linking of neutral inorganic alkoxide (TEOS) and ethanol precursors in the vesicles of surfactant molecules containing two polar head groups linked by hydrophobic alkyl chain, which rendered high specific surface area and pore volume of the resulting aminated PSiNs.

**Figure 4:**
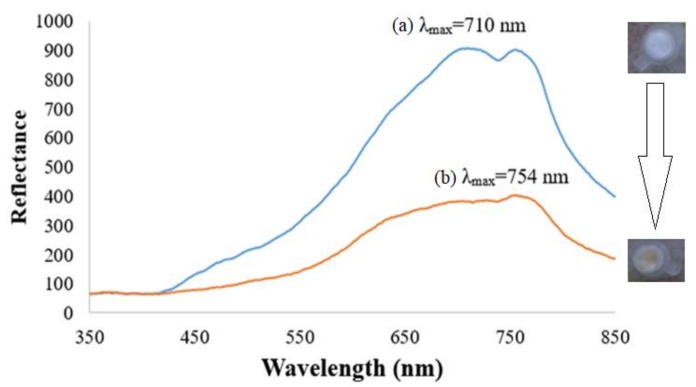
Dengue virus DNA biosensor reflectance spectra before (**a**) and after (**b**) hybridization with 1 × 10^−7^ µM target containing 1 mM ethyl piperidine substituted nickel(II) salphen complex at pH 7.0.

The square-planar piperidine side chain-grafted nickel(II) salphen complex is light yellow in colour prior to any DNA hybridization reaction. Upon hybridization between immobilized DNA probe and target DNA, the metal complex intercalated between bases of double-helical DNA by π-π staking interaction and exhibited a darker yellow background colour. Figure 4 shows the reflectance spectra of the dengue virus DNA biosensor based on aminated PSiNs DNA supporting matrix. The DNA probe-immobilized PSiNs demonstrated maximum reflectance intensity at 710 nm due to the light background colour of the APTS-modified PSiNs that have better light reflection characteristic compared to dull surfaces. After hybridization with DENV-2 cDNA and intercalation with nickel(II) Schiff base complex synthetic DNA marker, the optical DNA biosensor showed lower reflectance response (λ_max_ = 754 nm) ascribed to the colour change of the DNA biosensor to a darker yellow background, which has light absorbing characteristic. In addition, when the metal salphen complexes intercalated within dsDNA molecules, they caused base-pair separation and provided space in the double-helical chains of DNA. This lengthened the nucleic acid helix distance and that increased the dsDNA’s viscosity [23], which resulted in the decrement of reflectance response. The optical DNA biosensor showed the largest reflectance difference at a wavelength of 710 nm before and after hybridization with cDNA and intercalated with the metal complex intercalating agent, therefore it was chosen as the working wavelength in the subsequent DNA biosensor optimization experiments.

### 3.4. PSiNs, DNA Probe and Metal Complex Loading Optimizations

**Figure 5:**
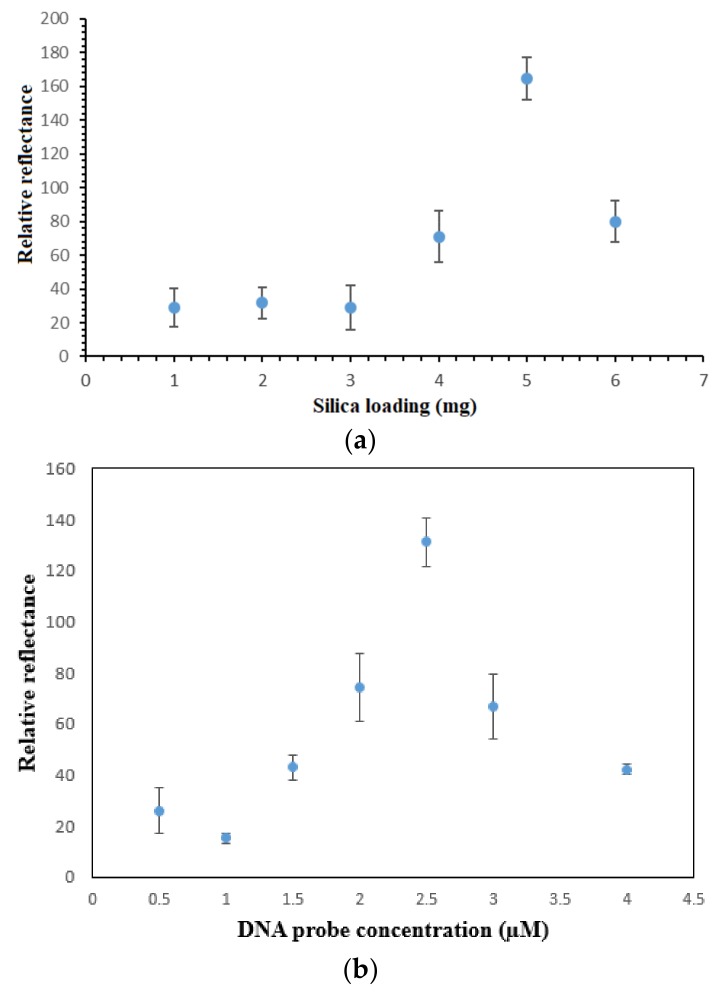

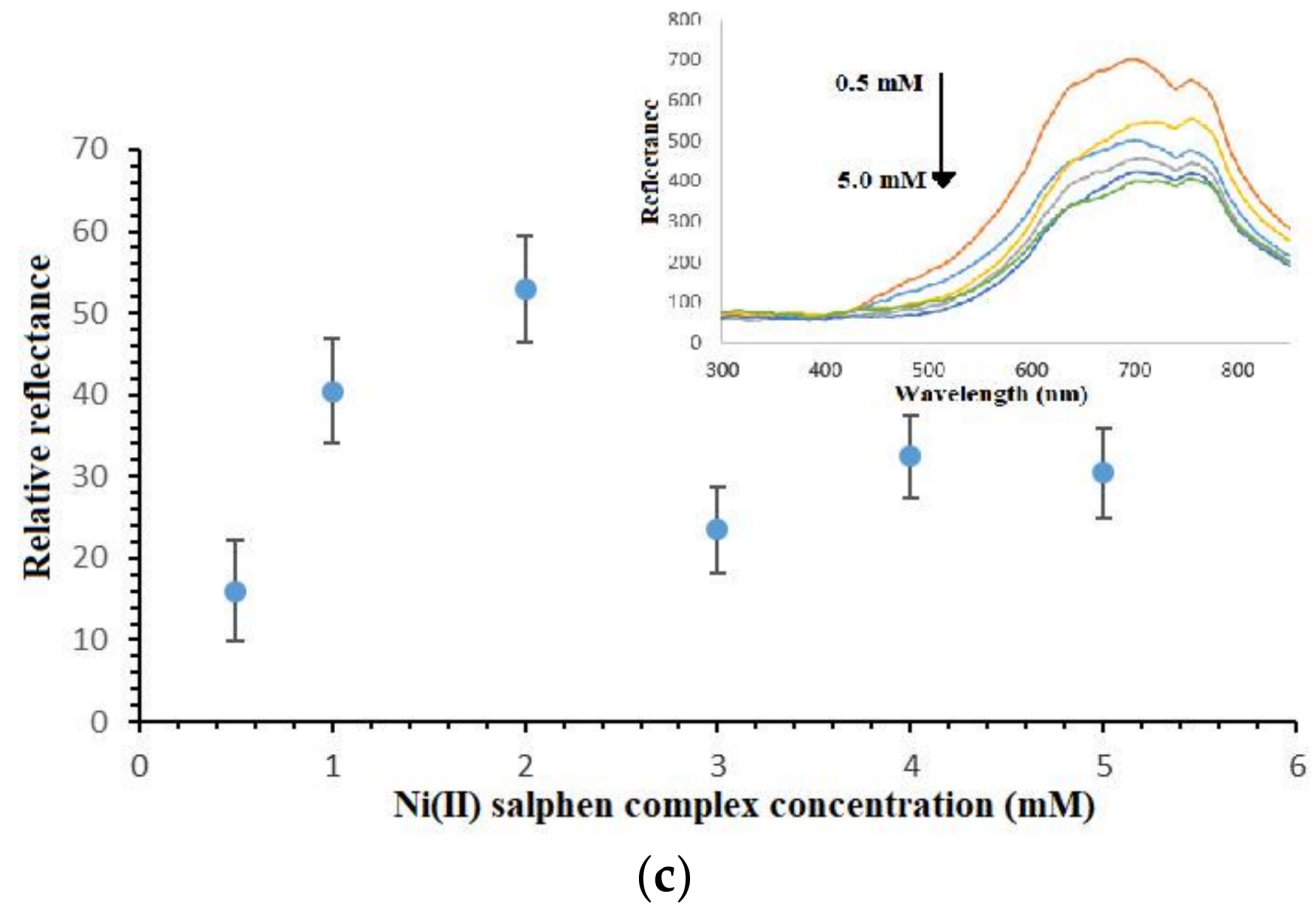
(**a**) Effect of PSiNs loading on the reflectometric response of the dengue virus DNA biosensor, (**b**) the reflectance response curve of the DNA biosensor with immobilized DNA probe between 0.5 µM and 4.0 µM towards the detection of 1 × 10^−7^ µM target DNA and (**c**) the effect of ethyl piperidine substituted nickel(II) salphen complex loading on the DNA hybridization response using 2.5 µM immobilized DNA probe and 1 mM metal complex binder.

Aminated PSiNs were employed as the DNA carrier matrix, which could provide large specific immobilization sites for DNA probe immobilization. Figure 5a depicts the effect of PSiNs loading on the reflectometric dengue virus DNA biosensor response. As the porous silica particles loading increased from 1 mg to 5 mg, the DNA biosensor response gradually increased due to the increasing number of DNA immobilization sites available for binding with a larger number of aminated DNA probes through covalent peptide linkages [24], thereby it promoted higher number of nickel(II) salphen complex intercalated within the DNA double helix. The DNA biosensor’s reflectance response declined when the PSiNs loaded above 5 mg. The high loading of supporting particles resulted in blocking of the specific immobilization active sites of the PSiNs and created a diffusion barrier to the movement of DNA fragments and metal complex intercalator to render specific DNA hybridization and intercalation reactions, and thus resulted in lower optical response.

The sensitivity of the DNA biosensor towards cDNA concentration is dependent on the method used for DNA probe immobilization and the optimum DNA probe amount immobilized on the matrix surface [25]. As Figure 5b indicates, the reflectance DNA biosensor response increased with the increase in the immobilized DNA probe quantity on the PSiNs up to 2.5 µM DNA probe, after which the DNA biosensor response declined from 3 µM DNA probe and onwards. Basically, the DNA hybridization reaction rate is directly proportional to the DNA probe loading. In a state of saturation, the PSiNs’ surface become densely packed with a layer of immobilized DNA probes, and the steric hindrance and repulsive electrostatic forces between negatively-charged immobilized DNA probes impede any further loading of higher DNA probe concentration. Thus, it reduces the DNA hybridization and metal complex intercalation reaction rates [26,27].

The as-prepared nickel(II) salphen complex with piperidine side chain is functionalized as the optical synthetic DNA binder, which could intercalate within bases of dsDNA by non-covalent aromatic-aromatic ring interaction between aromatic rings of square-planar metal complex and aromatic nucleobases in the DNA double helix. As the nickel(II) Schiff base complex intercalated into dsDNA, it gave a yellowish hue to the PSiNs-based DNA biosensor. The higher the concentration of metal complex intercalator (i.e., 0.5–2.0 mM) was introduced, the darker the yellow background colour developed to the DNA biosensor, therefore larger reflectance difference at 710 nm was obtained before and after the DNA biosensor hybridized with cDNA and intercalated with higher loading of the synthetic DNA marker (Figure 5c). However, given further increases in the intercalative complex concentration between 3.0 mM and 5.0 mM, the DNA biosensor did not show obvious colour change after hybridization with targeted DNA strands, and that no significant change in the relative reflectance signal was perceived. This was attributed to the saturation of immobilized dsDNA with metal complex synthetic DNA labels. The unavailability of the intercalation sites between base pairs of the immobilized DNAs prevented further interaction with the excess intercalator compound, hence giving relatively constant reflectance response.

### 3.5. Effect of Buffer Capacity and pH on the DNA Hybridization Reaction

**Figure 6:**
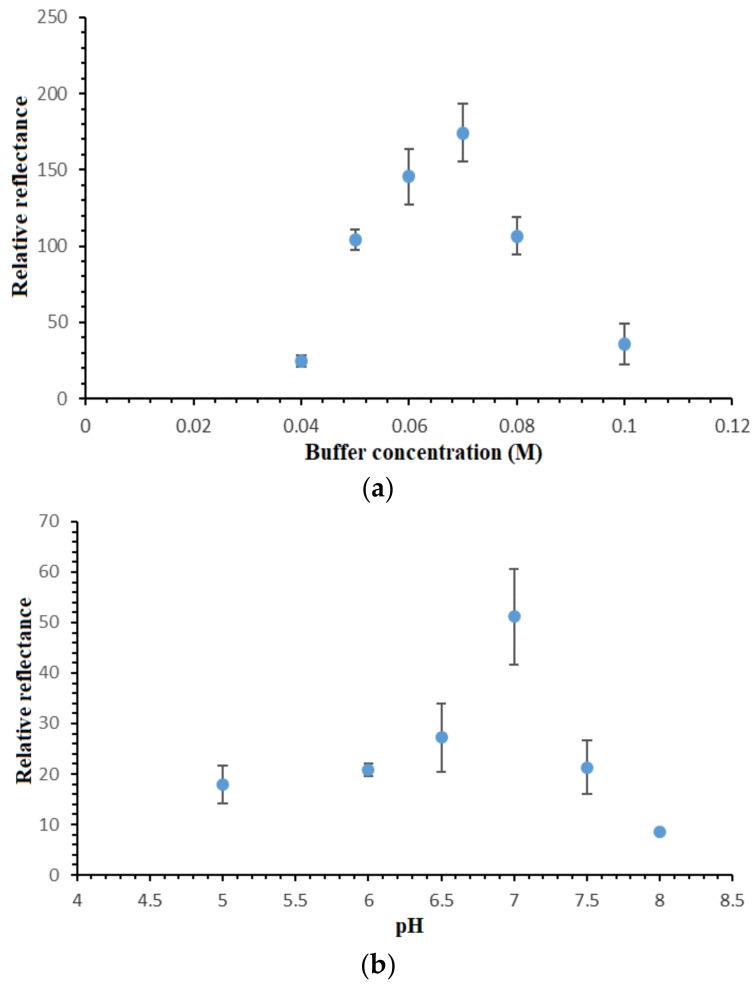
(**a**) Effect of Na-phosphate buffer capacity on the DNA hybridization response of the reflectance DNA biosensor at pH 7.0 using 1 × 10^−7^ µM cDNA and 2 mM metal complex marker and (**b**) pH profile of the optical DNA biosensor from pH 5.0–pH 8.0 using 0.07 M Na-phosphate buffer in the determination of 1 × 10^−7^ µM cDNA containing 2 mM synthetic DNA label.

Optimization of the buffer concentration is crucially needed in order to attain a suitable ionic surrounding for optimum DNA hybridization reaction to take place. Figure 6a shows the DNA biosensor’s relative reflectance changes in response to different Na-phosphate buffer concentrations. In view of the negatively-charged DNA phosphodiester chain, the presence of cation in the hybridization medium could react on the negatively-charged oligonucleotide and boost the DNA hybridization reaction rate [28]. By increasing the Na-phosphate buffer concentration from 0.04 M to 0.07 M, the DNA biosensor response increased proportionally due to the availability of the increasing amount of Na^+^ ions in neutralizing the DNA molecules, thus facilitating the DNA hybridization reaction between immobilized DNA probe and target DNA. Nevertheless, high buffer capacity i.e., above 0.07 M Na-phosphate buffer did not appear to reduce the electrostatic repulsion between negatively-charged phosphate groups of the immobilized dsDNA [29,30,31]. This reduced the DNA hybridization reaction rate, which was attributed to the increment in the steric repulsion between negatively-charged DNA sugar-phosphate backbone at high ionic strength DNA hybridization buffer. Since the use of 0.07 M Na-phosphate buffer showed the maximum DNA hybridization response, it was then utilized in the next DNA biosensor optimization studies. 

The pH profile of the reflectometric DNA biosensor is shown in Figure 6b. According to Hames and Higgins [32], solution pH is very influential on the DNA hybridization reaction rate. In acidic conditions, i.e., below pH 7.0, the protonization reaction of the phosphodiester chain in the DNA sugar-phosphate backbone reduced the DNA solubility in an aqueous environment, and it resulted in a sluggish DNA hybridization reaction rate between the immobilized DNA probe and cDNA [30]. At neutral pH, the protonization reaction on the DNA molecule phosphodiester chain was at a minimum level, and this promoted the DNA molecule solubility in water, hence increasing the DNA hybridization reaction rate. At alkaline pH, i.e., between pH 7.5 and pH 8.0, the basic pH broke up the hydrogen bonding between nitrogenous base pairs and denatured the helical dsDNA. As such, the optimum hybridization buffer pH was chosen as pH 7.0 for all DNA assays with the proposed optical DNA biosensor.

### 3.6. Determination of DNA Biosensor’s Hybridization Duration, Lifetime, Linear Range and Selectivity

**Figure 7:**
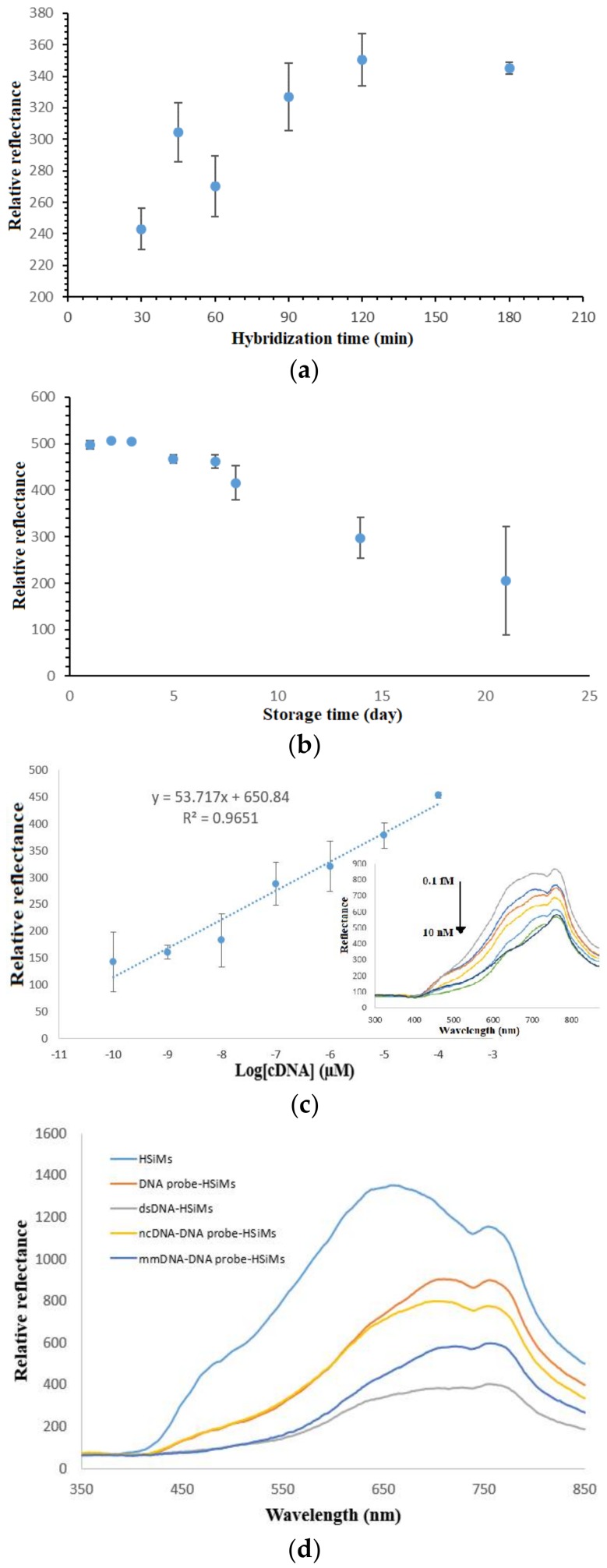
(**a**) DNA hybridization duration study carried with 0.07 M Na-phosphate buffer (pH 7.0) containing 1 × 10^−7^ µM cDNA and 2 mM synthetic DNA binder, (**b**) long-term stability profile of the optical dengue virus DNA biosensor in the quantification of 1 × 10^−7^ µM cDNA containing 2 mM metal complex at neutral pH, (**c**) dynamic linear response range of the optical dengue virus DNA biosensor (inset shows the corresponding reflectance spectra generated by the DNA biosensor in the cDNA concentration range of 1 × 10^−10^–1 × 10^−2^ µM and 2 mM ethyl piperidine substituted nickel(II) salphen complex DNA hybridization marker) and (**d**) the selectivity of the DNA biosensor towards target DNA, ncDNA and mismatch DNA (mmDNA) at 1 × 10^−8^ µM at pH 7.0.

The duration of the DNA biosensor hybridization is demonstrated in Figure 7a. The increment in the relative reflectance response from 30 min to 120 min implies the increasing DNA hybridization reaction rate occurred at the DNA biosensor surface, which allowed the subsequent metal complex intercalation reaction to take place. After that, no noticeable increment in the reflectance difference response was observed until 3 h of DNA hybridization time when the immobilized DNA probes on the PSiNs were entirely hybridized with target DNAs and reached a saturation state. Because 90 min of DNA hybridization time gave a detectable reflectance signal, thus the DNA biosensor response time was set at 90 min in the following analytical optimization works.

The long-term stability study of the DNA biosensor revealed the shelf life of the developed optical dengue virus DNA biosensor to be around one week. Based on the DNA biosensor reflectance response trends exhibited in Figure 7b, the reflectance DNA biosensor was capable of retaining about 92.8% of its initial response on day 7. Thereafter, the DNA biosensor response dropped to around 59.8% of its initial response on day 14 followed by 41.2% after three weeks of the experimental duration. The substantial decline in the DNA biosensor response after three weeks in storage was attributed the degradation of immobilized DNA probe on the porous silica particles, which hindered the formation of helical dsDNA structure.

Figure 7c represents the linear response range of the developed optical dengue virus DNA biosensor between 1 × 10^−10^ µM and 1 × 10^−4^ µM with correlation coefficient, R^2^ = 0.9651. The inset in Figure 5c shows the DNA biosensor reflectance response at 710 nm decreased proportionally with the increase in the dengue virus cDNA concentration from 1 × 10^−10^–1 × 10^−4^ µM due to the colour change of the DNA biosensor to a darker yellow background colour with the increasing target DNA concentration in the presence of constant intercalative complex concentration at 2 mM, and reaching a plateau response when the cDNA concentration was further added to 1 × 10^−2^ µM. This signifies the saturation of the immobilized DNA probe with cDNA. The limit of detection (LOD) of the DNA biosensor was determined as the cDNA concentration equivalent to the blank signal plus three times the standard deviation of blank was estimated at 0.2 aM cDNA. Blank refers to the biosensor that is free from any receptors and labels and giving maximum reflectance response at 710 nm for a consecutive series of individual measurements. The average reproducibility relative standard deviation (RSD) of each calibration point of the reflectometric DNA biosensor acquired, using three individual biosensors for each DNA concentration testing was calculated at 8.1%.

The selectivity of the DNA biosensor is imperative in sequence specific determination of target DNA strand. Figure 7d illustrates the selectivity of the DNA biosensor for hybridization with target DNA, non-target DNA and 1-base unpaired DNA. The APTS-modified PSiNs appeared to give the highest reflectance intensity, which was ascribed to its white background colour that reflected all the colours of the visible spectrum. After the PSiNs DNA carrier matrix was immobilized with a layer of dengue virus DNA probe, the slightly dull or rough surfaces reflected lower light intensity as light colour reflects better than dark colour. Upon testing with cDNA in the presence of metal complex oligonucleotide marker, the DNA biosensor revealed a drastic decrease in the reflectance signal at 710 nm as a yellow coloration developed on the DNA biosensor. Hybridization of the immobilized DNA probe with ncDNA showed a 10.3% decrement in the reflectance intensity as compared to the result obtained with cDNA due to the ncDNA bases, which rendered the partially hybridized DNA intercalated with lower amount of transition metal complex label. The unpaired DNA strand, however, showed a 67.2% drop in the reflectance response compared to the cDNA strand. This can be explained by the fact that the 1-base mismatched DNA is composed of 94% complementary base pairs. Thus, it can be deduced that the developed optical DNA biosensor is highly selective towards DENV-2 detection, being able to distinguish even a single base pair. 

### 3.7. Recovery Test

The described reflectance dengue virus DNA biosensor was applied to determine the known synthetic cDNA concentration spiked into non-invasive human bodily fluid samples such as urine and saliva, as dengue virus infection in human has been reported to be detected in both urine and saliva but not in plasma [33]. The reflectance response of saliva and urine samples obtained with the optical DNA biosensor without spiking of standard cDNA shows noise levels at maximum reflectance response at 710 nm were essentially negated. As the reflectance noise responses were converted to cDNA concentration in µM unit using the biosensor’s calibration curve equation, cDNA concentrations at extremely low level were obtained, and which were almost undetectable by the proposed DNA biosensor. Ideally, the recovery performance of the DNA biosensor is about 100%, whilst the allowable total error is ±20% for acceptable biosensor performance. Referring to Table 2, the recovery percentage range obtained by the proposed DNA biosensor is between 91.6% and 115%, thus it complies with the criterion for acceptable performance. The average recovery value obtained at close to 100% indicates high accuracy of the DNA biosensor for optical detection of dengue virus.

## 4. Conclusions

The reflectometric dengue virus DNA biosensor based on PSiNs DNA immobilization matrix and nickel(II) salphen complex optical DNA marker exhibited sensitive, selective and rapid screening of dengue viruses, which can be employed as a solution to existing analytical methods. The large reaction surface area of the nanosensing platforms enhanced the DNA hybridization reaction rate to give a lower detection limit and higher sensitivity. In addition, the proposed DNA analytical technique showed high promise for further transformation into a portable colorimetric nano-biosensor kit for in situ detection of the dengue virus in non-invasive human bodily fluids based on visual colour inspection.

## Figures and Tables

**Table 1:** The DNA oligonucleotide sequences used in the present research.

DNA	Base Sequences
DNA probe	5′-NH_2_-TTTTGTCCTGCTCTTCATTTAGGCTGGGTT-3′
cDNA	5′-AACCCAGCCTAAATGAAGAGCAGGACAAAA-3′
ncDNA	5′-AACGCCGATACCATTACTTATACCGCGACG-3′
1-base mismatched DNA	5′-AACCCATCCTAAATGAAGAGCAGGACAAAA-3′

**Table 2:** Recovery percentage of spiked cDNA concentration in saliva and urine samples by using the developed reflectance dengue virus DNA biosensor.

Samples	Spiked cDNA Concentration (µM) ^1^	Reflectance Response (a.u.)	cDNA Concentration Determined by DNA Biosensor (µM) ^2^	Recovery (%) ^3^
Saliva	0.00	1111.22 ± 83.32	7.65 × 10^−13^	-
	1.00 × 10^−4^	678.12 ± 14.26	9.80 × 10^−5^	98.0
	1.00 × 10^−5^	727.67 ± 12.37	1.13 × 10^−5^	113.0
	1.00 × 10^−7^	833.2 ± 3.52	1.15 × 10^−7^	115.0
Urine	0.00	1068.10 ± 89.13	7.65 × 10^−13^	-
	1.00 × 10^−4^	641.80 ± 29.34	9.66 × 10^−5^	96.6
	1.00 × 10^−5^	738.53 ± 63.89	9.97 × 10^−6^	99.7
	1.00 × 10^−7^	820.97 ± 44.61	9.16 × 10^−8^	91.6

^1^ The actual concentration of DENV-2 target DNA spiked into the human fluid samples, C; ^2^ The concentration of DENV-2 target DNA determined by the reflectometric DNA biosensor, C_s_; ^3^ The recovery percentage of spiked DENV-2 target DNA concentration by the optical DNA biosensors based on % Recovery = Cs/C × 100 %.

## Supplementary Materials

The following are available online at http://www.mdpi.com/1424-8220/18/4/1173/s1, Figure S1: ^1^H NMR spectrum (a) and ^13^C NMR spectrum (b) of the ethyl piperidine substituted nickel(II) salphen complex in DMSO-*d*_6_, Figure S2: Infra-red spectrum of the as-synthesized nickel(II) salphen complex with piperidine side chain, Figure S3: Mass spectrum for the nickel(II) salphen complex with piperidine side chain, Figure S4: UV-Vis spectra for the titration of ethyl piperidine substituted nickel(II) salphen (30 µM) complex binding to dsDNA using cDNA concentration from 6 × 10^−6^ M–1.8 × 10^−5^ M, Figure S5: The Scatchard plot linear binding.
