# Supplementary material for: Optical DNA Biosensor Based on Square-Planar Ethyl Piperidine Substituted Nickel(II) Salphen Complex for Dengue Virus Detection
Source: Sensors (Basel). 2018 Apr 12;18(4):1173. doi: 10.3390/s18041173 (PMC5948868; doi:10.3390/s18041173)
Supplement: Supplementary file 1 [file sensors-18-01173-s001.pdf]

Supplementary Material

a)

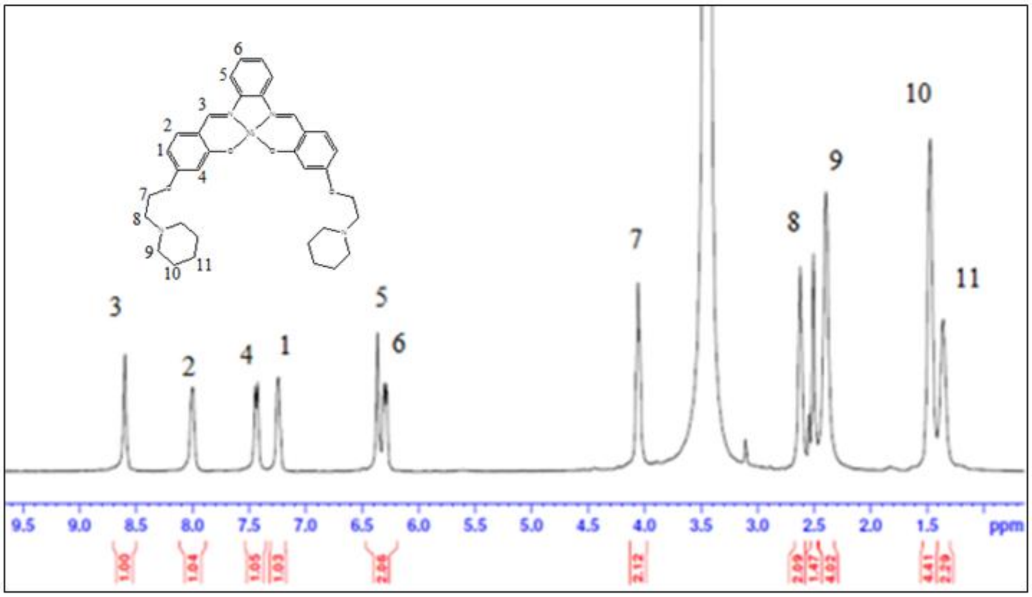

b)

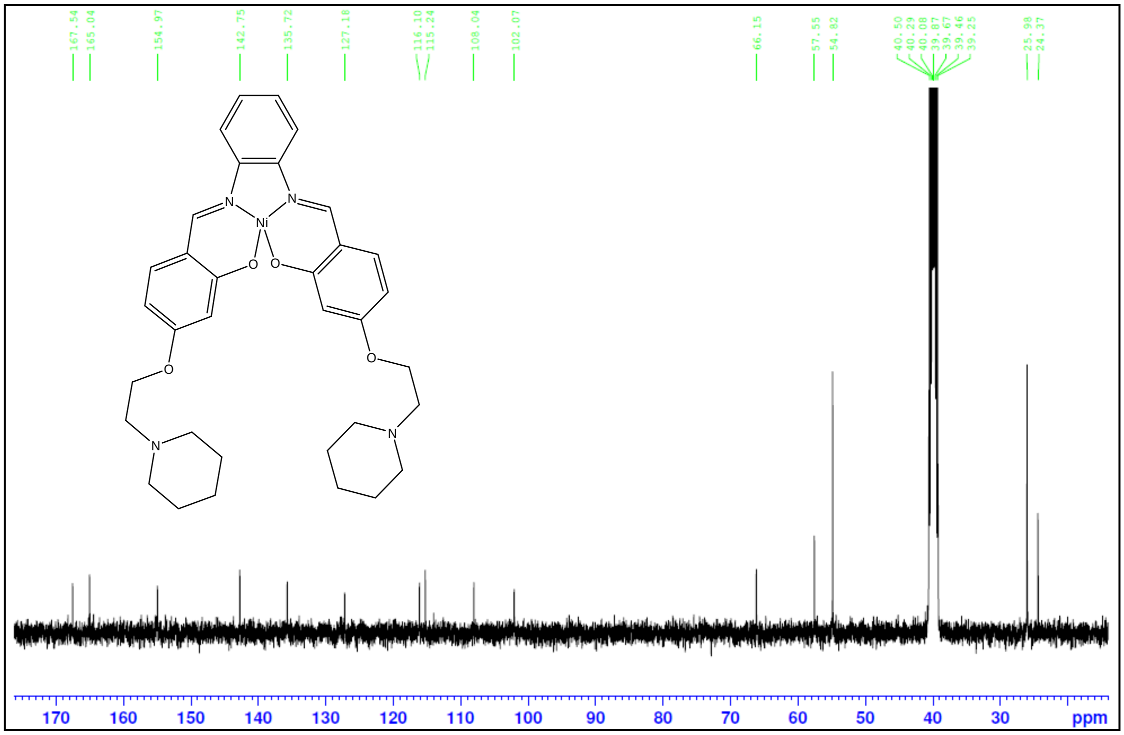

Figure S1. ^{1}H NMR spectrum (a) and ^{13}C NMR spectrum (b) of the ethyl piperidine substituted nickel(II) salphen complex in DMSO-d_6.

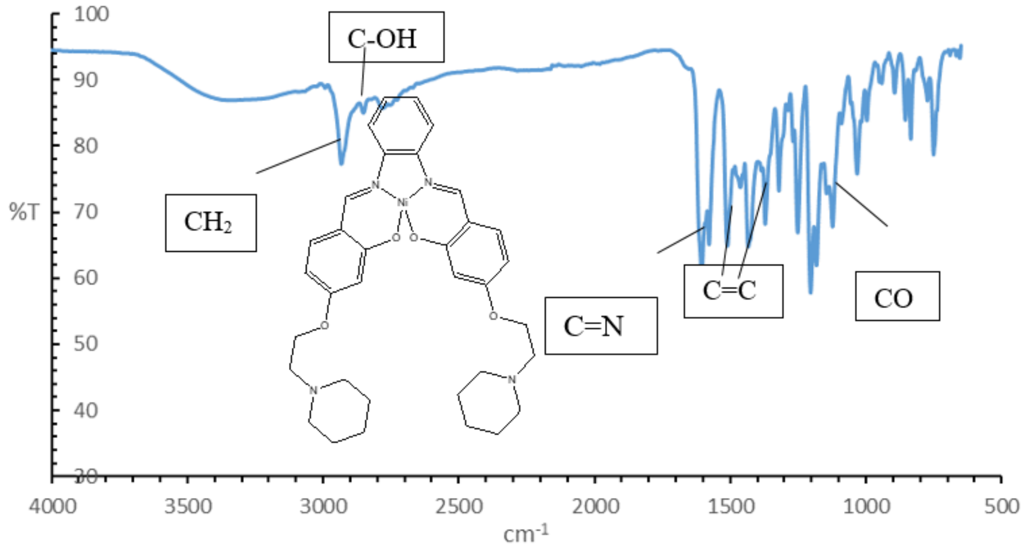

Figure S2. Infra-red spectrum of the as-synthesized nickel(II) salphen complex with piperidine side chain.

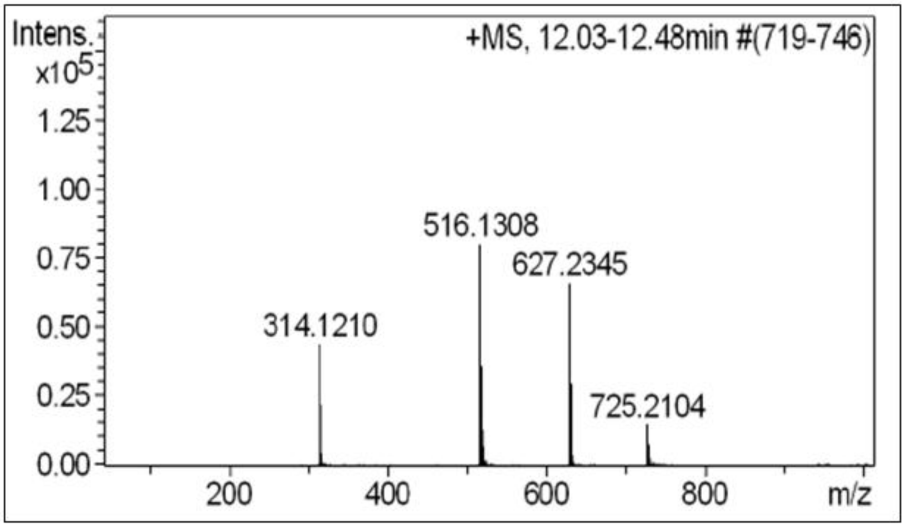

Figure S3. Mass spectrum for the nickel(II) salphen complex with piperidine side chain.

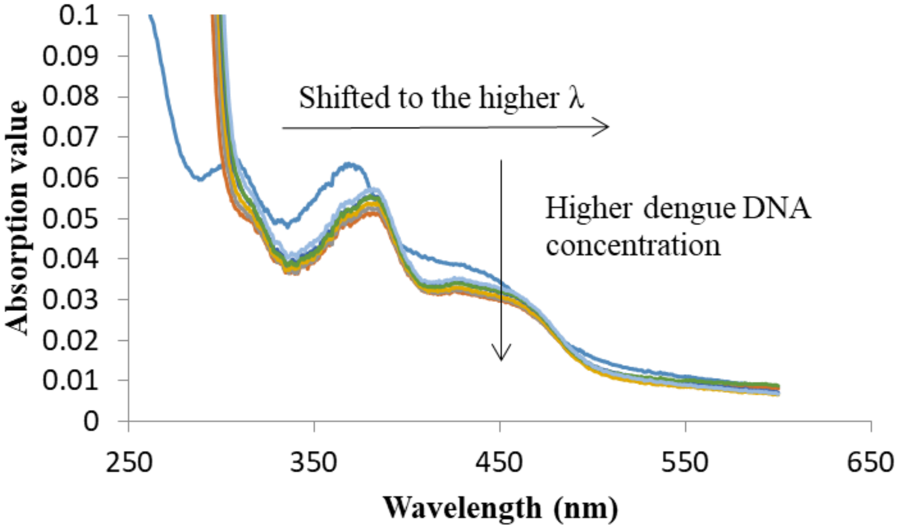

Figure S4. UV-Vis spectra for the titration of ethyl piperidine substituted nickel(II) salphen (30 μM) complex binding to dsDNA using cDNA concentration from 6×10^{-6} M-1.8×10^{-5} M.

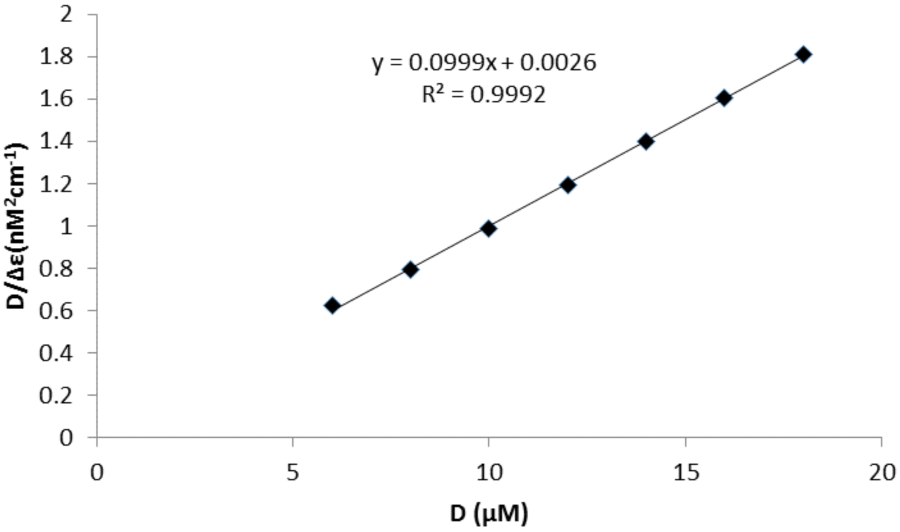

Figure S5. The Scatchard plot linear binding.
